# Oral condition of patients hospitalized for Covid-19 and its impact on quality of life

**DOI:** 10.1590/1807-3107bor-2025.vol39.009

**Published:** 2025-02-03

**Authors:** Lara Ribeiro Feitosa DUAILIBE, Laise Nunes RODRIGUES, Alanna Barros de ARRUDA, Robinson SABINO-SILVA, Rayenne Augusta Mota FERREIRA, Rudys Rodolfo de Jesus TAVAREZ, Cyrene Piazera Silva COSTA, Meire Coelho FERREIRA

**Affiliations:** (a) Universidade Ceuma – Uniceuma, Graduate Program in Dentistry, Department of Dentistry, São Luís, MA, Brazil.; (b) Universidade Federal de Uberlância – UFU, Institute of Biomedical Sciences, Department of Physiology, Uberlândia, MG, Brazil.

**Keywords:** SARS-CoV-2, COVID-19, Intensive Care Units, Oral Health, Patient-Reported Outcome Measures, Quality of Life

## Abstract

The aim of this study was to assess the oral condition of individuals diagnosed with COVID-19 and its impact on their quality of life. The cross-sectional study participants were patients with or without a diagnosis of COVID-19, on room air, and conscious, admitted to the ICUs and wards of Public Hospital Units in São Luís, Maranhão, Brazil. The data collected included: demographic information, length of stay, comorbidities, and type of diet, obtained from medical records; Oral Health-Related Quality of Life (OHRQoL) [Oral Health Impact Profile (OHIP-14)]questions patients were asked; oral health (measured by the Bedside Oral Exam Scale); oral hygiene status (assessed by the Oral Hygiene Index - Simplified and lingual: degree of lingual coating); and salivary flow. The prevalence of COVID-19 was associated with gender (p = 0.038), with a higher incidence observed in male patients (61.9%). Moreover, there was a correlation between the hospitalization sector (p = 0.037) and the frequency of ICU admissions (53.7%). The prevalence of comorbidities was comparable between the two groups. Relative to oral health, 53% of individuals with confirmed COVID-19 had moderate oral health, while 9% exhibited poor oral health. The prevalence of hyposalivation was higher in the group with a confirmed diagnosis of COVID-19. The quality of life of individuals with confirmed COVID-19 was most significantly impacted by moderate to severe oral health concerns. The most significant alteration in oral health was a reduction in salivary flow, negatively impacting the quality of life of individuals hospitalized for COVID-19 complications.

## Introduction

Oral diseases can be conceptualized as a category of non-lethal chronic processes. However, in their acute phase, they manifest as symptomatic consequences, such as pain and discomfort; functional impairments, such as speech, swallowing, and chewing; and psychosocial consequences that have significant implications for people’s daily lives.^1^ In hospitalized patients, compromised oral health, aggravated by limited oral hygiene, has been observed to reflect on the general clinical condition of the patient, resulting in prolonged hospital stays and compromised quality of life.^2^

Oral health-related quality of life (OHRQoL) is a multidimensional construct that reflects an individual’s comfort when eating, sleeping, and engaging in social interactions. It also reflects their self-esteem, satisfaction with oral health, and other factors.^3^ This indicates that when oral health is compromised, it can impact various aspects of life, including function, appearance, and interpersonal relationships. The OHRQoL represents the assessment of oral health impairments experienced by individuals, serving as a supplementary measure to the clinical diagnosis made by healthcare professionals.^4^

In the case of individuals hospitalized for complications associated with SARS-CoV-2 virus, in addition to the systemic condition primarily affecting the respiratory system,^5^ there is presence of oral manifestations such as decreased salivary flow, soft tissue lesions, opportunistic infections, and taste deficiencies.^6-8^ A lack of oral hygiene and salivary flow, whether due to negligence or other causes, can contribute to the development of co-infections such as oral candidiasis,^8^ gingivitis,^9^ and dental caries.^10^

To date, only a limited number of studies have examined the construct of quality of very rapid self-assessment of breathing (OHRQoL) within the context of the ongoing Coronavirus Disease 2019 (COVID-19) pandemic. Among these studies, one investigated the impact of pain resulting from temporomandibular disorders (TMD) before and during the COVID-19 pandemic^11^ and another examined the impact of dental pain on people’s daily lives during the period of social distancing.^2^ However, to date, no studies have been found in the databases used to conduct the bibliographic search performed for the purpose of evaluating the impact of the clinical oral condition on quality of life of individuals hospitalized for COVID-19.

This study addressed this gap by examining the oral health of hospitalized patients with COVID-19 and its impact on their quality of life. Understanding this relationship is crucial as it can provide insights into the broader implications of oral health on patient outcomes during hospitalization. The findings could inform those who engage in healthcare practices and establish policies, with the aim of improving oral hygiene and overall care for hospitalized patients, potentially reducing hospital stays and enhancing their quality of life. Furthermore, this study could pave the way for further research exploring the interconnectedness of oral and systemic health, particularly within the context of infectious diseases such as COVID-19. Therefore, the aim of this study was to assess the oral health of individuals hospitalized for COVID-19 and its impact on their quality of life.

## Methods

The research was approved by the Research Ethics Committee of Ceuma University (4.610.070) and was conducted as a cross-sectional study involving individuals who were hospitalized in São Luís, Maranhão, in the northeast region of Brazil. The eligibility criteria for the study participants were patients with or without diagnosis of COVID-19, with room air and conscious, admitted to the intensive care units (ICUs) and wards of public hospital units in São Luís, Maranhão, Brazil.

For this study, a sample power calculation was performed considering two groups: a group without COVID-19 with 115 participants and a group with COVID-19 with 167 participants. With an effect size (d) of 0.8 and a significance level (αα) of 0.05, the sample power was calculated to be 0.99 using the G*Power 3.1 program.

The following data were extracted from the medical and dental records of patients hospitalized with or without a diagnosis of COVID-19: demographic characteristics, hospitalization sector, length of stay, comorbidities, type of diet (oral or enteral), oral condition (measured by the Bedside Oral Scale Exam [BOE]), oral hygiene (tooth hygiene and tongue hygiene [degree of lingual coating]), and salivary flow.

The BOE encompasses an assessment of the following areas: swallowing, lips, tongue, saliva, mucosa, gums, teeth or prostheses, and odor. Each item is categorized into one of three levels of dysfunction: normal (score 1), moderate (score 2), and severe (score 3). Based on the total score, oral health is classified as good (total score of 8 to 10 points), moderate (total score of 11 to 14 points), or poor (total score of 15 to 24 points)^29^ (Table 1).

**Table 1:** Numerical and descriptive classifications by category of the Beside Oral Exam (BOE) Scale.

Category	Numerical and descriptive classifications		
	1	two	3
	Normal	Moderate dysfunction	Severe dysfunction
Deglutition	Normal Swallowing	Pain or difficulty swallowing	Unable to swallow
			(intubated)
Lips	Soft and pink	Dry or cracked.	Ulceration or bleeding.
Language	Pink, moist and papillae present	Loss of papillae, or covered by shiny papillae, red or not	Blistered, cracked or bleeding.
Spittle	Hydrated	Thick or sticky	Absent
Mucosa	Pink and wet	Reddish without ulcers	Ulcers and/or bleeding
Gum	Pink and firm	Edema, with or without redness; with or without bleeding	Bleeds easily
Teeth or dentures	Clean or toothless	Local dental calculations	Generalized dental calculus and/or caries
		(between teeth)	
Odor	Normal	Slight or moderate unpleasant odor	Strong unpleasant odor

The subjects’ dental hygiene was evaluated using the Oral Hygiene Index - Simplified (OHI-S)^13^. A total of six teeth namely Teeth 16, 11, 26, and 31 were examined buccally, while teeth 36 and 46 were examined lingually. In the absence of any of these teeth, the adjacent tooth could be used as a substitute. The plaque scores were as follows: 0 (absence of plaque); 1 (plaque covering less than one-third of the tooth surface); 2 (plaque covering more than one-third but less than two-thirds of the tooth surface); 3 (plaque covering two-thirds of the tooth surface); and X (no index tooth or substitute). The OHI-S was calculated by adding up the plaque scores of each tooth and dividing the result by six. The resulting value was then classified as follows: scores of 0-1.2 are indicative of good oral hygiene, while scores of 1.3-3 are indicative of fair oral hygiene, and scores of > 3.1 are indicative of poor oral hygiene.

Lingual hygiene was evaluated using the Lingual Saburra Degree (LSD)^14^. The data were classified as follows: The scale ranged from 0 (absence of saburra), 1 (light saburra on the posterior third of the tongue), 2 (light saburra on the posterior and middle thirds of the tongue), 3 (moderate saburra on the posterior third of the tongue), 4 (moderate saburra on the posterior and middle thirds of the tongue) and 5 (moderate saburra on the posterior, middle, and anterior thirds of the tongue). Mild saburra was defined as the presence of visible lingual papillae, whereas moderate saburra was characterized by the absence of these papillae, with the lingual papillae covered by the saburra.

In order to quantify the salivary flow of conscious patients, they were seated comfortably, and the procedure commenced after they had swallowed once in order to remove the saliva present in the oral cavity (zero time). The patient was instructed to refrain from swallowing or chewing during the collection period, which lasted seven minutes. Saliva was collected in polypropylene tubes. Subsequently, the salivary flow (ml/min) was quantified by assessing the weight of the empty tube, with a ratio of 1 mg equal to 1 ul.^15^ The results were interpreted in accordance with the criteria established by Flink,^16^ whereby a salivary flow rate exceeding 1.0 ml/min is indicative of normal salivary function, a rate between 0.7 and 0.99 ml/min is indicative of hyposalivation, and a rate below 0.7 ml/min is indicative of xerostomia.

The Brazilian version of the Oral Health Impact Profile (OHIP-14)^17^ was used to assess the impact of oral conditions on the quality of life of patients over the past six months. The instrument consists of seven domains, each comprising two items: functional limitation, physical pain, psychological discomfort, physical disability, psychological disability, social disability, and disability. The impact frequency was gauged on a Likert scale, with the following response categories: The response categories were as follows: never (0), rarely (1), sometimes (2), often (3), and always (4). The total OHIP-14 score was calculated using the additive method, with a potential range of 0 to 56. A higher score indicated a greater negative impact on OHRQoL.

The data were submitted to descriptive and inferential statistical analyses. Then they were evaluated for distribution and homogeneity of variance using the Kolmogorov-Smirnov and Levene tests, respectively. A chi-square test was used to evaluate the comparative impact of the independent variables on the two groups (with and without a history of SARS-CoV-2 infection). To assess the mean score for each domain and the total OHIP-14 score, a Mann-Whitney test was used. The significance level adopted was 5%. All statistical analyses were conducted using the Statistical Package for Social Sciences (SPSS, version 21.0, IBM Corporation, Armonk, USA).

## Results

Among patients hospitalized for COVID-19, 61.9% were male and 53.7% were admitted to the intensive care unit (ICU). The majority of patients remained in hospital for a period of between one and seven days, and the prevalence of comorbidities was comparable between groups (Table 2).

**Table 2:** Demographic characteristics, related to hospitalization and comorbidities of patients hospitalized with and without COVID-19 (n = 483).

Variables	Without COVID-19	With COVID-19	p-value^***^
	n (%)	n (%)	
Sex			
Masculine	113 (52.6)	166 (61.9)	0.038
Feminine	102 (47.4)	102 (38.1)	
Age range (years)			
0–59	101 (47)	126 (47)	0.993
60–97	114 (53)	143 (53)	
Sector			
Nursery	120 (55.8)	124 (46.3)	0.037
ICU*	95 (44.2)	144 (53.7)	
Hospitalization days			
1–7	153 (71.2)	191 (71.3)	
8–14	46 (21.4)	59 (22.0)	0.976
15–27	16 (7.4)	18 (6.7)	
Comorbidities			
No	46 (21.4)	71(26.5)	0.194
Yes	169 (78.6)	197 (73.5)	
Diabetes Mellitus			
No	122(56.7)	166 (61.9)	0.247
Yes	93 (43.3)	102 (38.1)	
Hypertension			
No	78(36.3)	105 (39.2)	0.514
Yes	137 (63.7)	163 (60.8)	
Obesity			
No	206 (95.8)	251 (93.7)	0.296
Yes	9 (4.2)	17 (6.3)	
DCR**			
No	189 (87.9)	247 ( 92.2)	0.117
Yes	26 (12.1)	21 (7.8)	
Cardiopathies			
No	182 (84.7)	232 (86.6)	0.550
Yes	33 (15.3)	36 (13.4)	
Down’s syndrome			
No	212 (98.6)	266 (99.3)	0.660 φ
Yes	3 (1.4)	2 (0.7)	
Asthma			
No	212 (98.6)	265 (98.9)	1,000 φ
Yes	3 (1.4)	3 (1.1)	
Neuro sequelae			
No	202 (94.0)	256 (95.5)	0.439
Yes	13 (6.0)	12 (4.5)	
Alzheimer’s disease			
No	207 (96.3)	259 (96.6)	0.830
Yes	8 (3.7)	9 (3.4)	
Sickle cell anemia			
No	212 (98.6)	265 (98.9)	1,000 φ
Yes	3 (1.4)	3 (1.1)	

*ICU: Intensive care unit; **CKD: Chronic kidney diseases; ^***^Chi -square test.

Table 3 illustrates the prevalence of oral conditions among individuals hospitalized with a diagnosis of COVID-19. A significant association was observed between BOE and a diagnosis of COVID-19 (p < 0.001). Among individuals with COVID-19, 53% exhibited a moderate oral condition, while 9% had a deteriorated oral condition. Among the oral aspects of which the BOE consists, hyposalivation was observed in 81.3% of individuals hospitalized for complications associated with the SARS-CoV-2 virus.

**Table 3:** Oral conditions of people hospitalized with and without COVID-19 (n = 483).

Variables	Without COVID-19	With COVID-19	p-value*
	n (%)	n (%)	
Oral health (measured by BOE)			
Good	157 (73.0)	102 (38.1)	< 0.001
Moderate	58 (27.0)	142 (53.0)	
Poor	0 (0.0)	24 (9.0)	
OHI-S			
Good (0–1.2)	179 (83.3)	225 (84)	0.827
Fair (1.3–3)	27 (12.6)	29 (10.8)	
Poor (> 3.1)	9 (4.2)	14 (5.2)	
Degree of tongue saburra			
Absence of saburra	50 (23.3)	48 (17.9)	0.209
Mild (back 1/3 of tongue)	70 (32.6)	72 (26.9)	
Light (rear/middle 1/3)	39 (18.1)	50 (18.7)	
Moderate (later 1/3)	16 (7.4)	28 (10.4)	
Moderate (later 1/3/medium)	25 (11.6)	44 (16.4)	
Moderate (1/3 posterior/medium/anterior)	15 (7.0)	26 (9.7)	
Salivary flow			
Normal	51 (23.7)	44 (16.4)	0.159
Xerostomia	0 (0.0)	1 (0.4)	
Hyposalivation	161 (74.9)	218 (81.3)	
Sialorrhea	3 (1.4)	5 (1.9)	
Diet			
Oral	196 (91.2)	243 (90.7)	0.852
Enteral	19 (8.8)	25 (9.3)	

^*^Chi -square test.

The impact of moderate/poor oral health (BOE with a score of 11 –24) on the quality of life of hospitalized patients revealed that those with a diagnosis of COVID-19 exhibited a more pronounced impairment than those without this diagnosis (p < 0.001). The impact was verified by means of the domains “psychological discomfort”, “social disability”, “disability”, and “total score” (p = 0.001; p = 0.017; p < 0.001 and p = 0.014, respectively) (Table 4).

**Table 4:** Impact of oral health, measured by BOE, on the quality of life of people hospitalized with and without COVID-19 (n = 483).

OHIP-14 Domains	Good oral health (BOE 8 to 10)			Moderate to poor oral health (BOE 11 to 24)		
	Withour COVID-19	With COVID-19	p-value*	Withour COVID-19	With COVID-19	p-value*
	(n = 157)	(n = 102)		(n = 58)	(n = 166)	
	Average (SD)	Average (SD)		Average (SD)	Average (SD)	
	Median; 25%-75%	Median; 25%-75%		Median; 25%-75%	Median; 25%-75%	
Functional Limitation	1.20 (1.11)	1.47 (1.30)	0.110	1.21 (1.04)	1.43 (1.18)	0.207
	1.00; 1.02–1.37	1.50; 1.21–1.73		1.00; 0.93–1.48	1.00; 1.25–1.61	
Physical pain	1.55 (1.34)	1.85 (1.16)	0.007	1.60 (1.40)	1.83 (1.13)	0.052
	2.00; 1.34–1.76	2.00; 1.62–2.08		2.00; 1.24–1.97	2.00; 1.65–2.00	
Psychological discomfort	0.68 (1.35)	1.23 (1.01)	<0.001	0.76 (1.54)	1.05 (0.99)	0.001
	1.81; 0.47–0.89	1.00; 1.03–1.42		0.00; 0.35–1.16	1.00; 0.90–1.21	
Physical disability	1.03 (1.16)	1.04 (1.02)	0.652	0.98 (1.16)	1.19 (1.04)	0.087
	1.00; 0.85–1.21	1.00; 0.84–1.24		1.00; 0.68–1.29	1.00; 1.03–1.35	
Psychological disability	1.27 (1.55)	0.98 (1.00)	0.579	1.38 (1.59)	0.99 (0.93)	0.349
	1.00; 1.02–1.51	1.00; 0.78–1.18		1.00; 0.96–1.80	1.00; 0.84–1.13	
Social disability	0.54 (0.90)	0.64 (0.76)	0.064	0.41 (0.80)	0.67 (0.86)	0.017
	0.00; 0.40–0.68	0.50; 0.49–0.79		0.00; 0.20–0.62	0.00; 0.54–0.80	
Deficiency	0.69 (1.39)	0.86 (1.00)	<0.001	0.60 (1.44)	0.77 (0.90)	<0.001
	0.00; 0.48–0.91	1.00; 0.67–1.06		0.00; 0.23–0.98	1.00; 0.63–0.91	
Total score	6.97 (6.11)	8.07 (4.58)	0.002	6.95 (6.60)	7.93 (4.88)	0.014
	5.00; 6.00 to 7.93	7.00; 7.17–8.97		5.00; 5.21–8.68	7.50; 7.19–8.68	

*Mann-Whitney test.

## Discussion

The primary findings of the study indicated a higher prevalence of COVID-19 among male patients and those requiring intensive care unit (ICU) admission. The BOE assessment revealed a correlation between oral health and the diagnosis of COVID-19. Specifically, individuals diagnosed with the virus exhibited poorer oral health outcomes. The prevalence of hyposalivation was higher among individuals with a confirmed diagnosis of COVID-19 than among those hospitalized for other medical conditions. With regard to the impact on quality of life, moderate and poor oral health significantly compromised those infected with the virus.

With regard to the preponderance of cases among men, a systematic review by Fang^18^ indicated that this demographic factor exhibited a greater severity of disease. Bourgonje^19^ posited that the greater involvement of men by the disease may be attributed to differences in exposure to the virus, smoking behavior, lifestyle, and chromosomal expression. Angiotensin-converting enzyme 2 (ACE2), ACE2 expression in testicular tissue, regulation of the immune system by sex hormones, and sex differences in regulation of the renin-angiotensin-aldosterone system (RAAS) are among the factors that may contribute to this phenomenon.

With regard to comorbidities, the most prevalent were type 2 diabetes mellitus (DM-2), systemic arterial hypertension (SAH), and other heart diseases for both study groups. The higher frequency of these three comorbidities in individuals with COVID-19 was consistent with the findings of a retrospective cohort study conducted using a sample of medical records of hospitalized patients in Wuhan, China.^5^ These comorbidities were associated with severe forms of the disease.^20^ Furthermore, they are predictors of mortality from the disease when present in conjunction with age, secondary infection, and elevated blood inflammatory markers.^21^

The hospitalization sectors found to be significantly associated with the novel COVID-19 were the intensive care units, in which a notable number of individuals with confirmed or suspected cases of the disease received treatment. This further substantiated the role of comorbidities as explanatory factors for the severe form of the disease, which in turn needed more intensive care during hospitalization.^22^

The act of hospitalization, particularly within the context of intensive care units (ICUs), renders patients susceptible to a multitude of external and internal threats to their oral health.^23^ A variety of oral health issues that pose a threat to life or result in long-term complications may be present.^8^ Infection with the acute respiratory syndrome coronavirus 2 (SARS-CoV-2) resulted in oral manifestations affecting the buccal mucosa, salivary glands, and/or sensory elements. These oral manifestations resulted from the presence of SARS-CoV-2 due to the high expression of ACE2 receptors in oral epithelial cells and interactions between drugs used in the treatment of COVID-19.^24,25^

The presence of SARS-CoV-2 in the epithelial cells of the salivary glands can trigger an inflammatory response by initiating the replication process and cell lysis, which ultimately leads to the destruction of the glandular tissue. Consequently, in an attempt to repair the inflammatory damage, fibroblast proliferation and the formation of fibrous connective tissue occur, which results in a decrease in the immune reaction.?/response?/ This repair process can result in dysfunctions of the salivary glands, manifesting as a decrease in salivary flow, chronic sialadenitis, and infections.^26^ However, in the present study, hyposalivation was observed in the majority of individuals with COVID-19, as well as in patients hospitalized for other reasons. In patients admitted to the ICUs, low salivary flow, reduced natural cleansing of the oral cavity provided by mastication and movement of the tongue and cheeks, coupled with poor oral hygiene, facilitate the growth and formation of pathogenic bacterial biofilms on the dental surface and on the dorsum of the tongue.

It is therefore imperative that an oral hygiene protocol be established for ICU patients in order to prevent oral complications that may result in a deterioration of the patient’s overall clinical condition.^27^ A meticulous examination of the oral condition is imperative to forestall the onset of oral infections. The BOE is the instrument recommended for the oral evaluation of patients hospitalized in ICUs. It assesses a range of oral structures and functions, including swallowing, lips, tongue, saliva, mucosa, gums, teeth or prostheses, and odor.^28^ In the present study, individuals hospitalized in the hospital units researched were evaluated using the BOE. The results indicated that all those with poor oral health were part of the group with confirmed cases of COVID-19.

Anosmia and ageusia are symptoms frequently reported in the initial phase of COVID-19, which facilitates its diagnosis. As taste is the primary stimulant for salivary secretion, its absence may indicate the presence of hyposalivation and xerostomia,^29^thereby signifying the need for increasingly efficient oral care.

Given that oral alterations such as tongue coating and low salivary flow are known to cause considerable discomfort to hospitalized patients and to predispose them to the development of periodontal disease and dental caries, it is imperative that the OHRQoL of these individuals must be assessed as soon as feasible.

In this study, the negative impact on quality of life was significantly higher for patients with moderate or poor oral health, who had been diagnosed with COIVD-19 than for patients without the infection. This impact was observed by means of the domains “psychological discomfort,” indicating concern and feelings of stress; “social disability,” represented by irritation; and “disability,” and by the feeling that life had become worse.

To date, only studies that have evaluated OHRQoL during the ongoing pandemic have explored the impact of the virus on symptoms related to TMD and teeth. The study evaluating the impact of TMD pain on the quality of life of women found that this pain did not worsen with the advent of the pandemic, nor did it exert any influence on OHRQoL.^11^ Conversely, the duration and intensity of dental pain were identified as significant explanatory factors that influenced the impact on the quality of life of individuals who sought care at a tertiary dental care center during the course of the pandemic.^12^ In contrast to the aforementioned studies, the present study used a clinical diagnosis of the oral condition of individuals hospitalized for complications related to the SARS-CoV-2 and its impact on quality of life.

One limitation of this study is the relatively small sample size, which may affect the generalizability of the findings to broader populations. Additionally, the cross-sectional design limits the ability to establish causality between COVID-19 and oral health outcomes. The reliance on self-reported measures for some aspects of oral health may also introduce bias. Furthermore, the study was conducted within a single healthcare setting, which may not capture variations in oral health care practices and patient demographics across different regions or healthcare systems.

Despite these limitations, the study has several strengths. It addressed a significant gap in the literature by exploring the impact of oral health on quality of life in patients hospitalized with COVID-19, a topic that has received limited attention. The use of a comprehensive oral health evaluation tool (BOE) allowed for a detailed assessment of various oral health parameters, providing a robust measure of oral health status. By highlighting the prevalence of poor oral health and its association with COVID-19, the study underscored the importance of integrating oral health care into the overall management of hospitalized patients. Moreover, the findings emphasize the need for more intensive oral care protocols in ICU settings, which could improve patient outcomes and quality of life. The study also contributes to the broader understanding of patient-reported outcome measures (PROMs) in this population, potentially influencing patient-centered care practices among dentists and other healthcare professionals.

## Conclusion

The study found a significant association between oral health and a diagnosis of COVID-19. Furthermore, the study revealed that moderate to poor oral health significantly impaired the quality of life of patients hospitalized with COVID-19 compared with patients without the infection. These results underscore the critical impact of oral health on the overall well-being and quality of life of patients hospitalized with COVID-19, emphasizing the need for comprehensive oral care in this population.

The findings of this study reinforce the fundamental role of the dentist in the multidisciplinary team involved in hospital patient care. It is recommended that professional oral clinical evaluation, in conjunction with the patient’s perception of impact, be established as a protocol of conduct. This protocol will serve as the basis for an overall view of the patient’s health status, enabling the health team involved in the care to establish behaviors that may lead to the remission of inflammatory and infectious processes, and the restoration of a general health condition.
